# A Model-Based Approach for Bridging Virtual and Physical Sensor Nodes in a Hybrid Simulation Framework

**DOI:** 10.3390/s140611070

**Published:** 2014-06-23

**Authors:** Mohammad Mozumdar, Zhen Yu Song, Luciano Lavagno, Alberto L. Sangiovanni-Vincentelli

**Affiliations:** 1 Electrical Engineering, California State University Long Beach, 1250 N. Bellflower Blvd., Long Beach, CA 90840, USA; 2 Department of Electronics, Politecnico di Torino, Corso Duca degli Abruzzi 24, Turin 10129, Italy; E-Mails: song@ismb.it (Z.Y.S.); luciano.lavagno@polito.it (L.L.); 3 EECS Department, University of California at Berkeley, Cory Hall, Berkeley, CA 94720, USA; E-Mail: alberto@eecs.berkeley.edu

**Keywords:** model based design, wireless sensor networks, hardware-in-the-loop simulation

## Abstract

The Model Based Design (MBD) approach is a popular trend to speed up application development of embedded systems, which uses high-level abstractions to capture functional requirements in an executable manner, and which automates implementation code generation. Wireless Sensor Networks (WSNs) are an emerging very promising application area for embedded systems. However, there is a lack of tools in this area, which would allow an application developer to model a WSN application by using high level abstractions, simulate it mapped to a multi-node scenario for functional analysis, and finally use the refined model to automatically generate code for different WSN platforms. Motivated by this idea, in this paper we present a hybrid simulation framework that not only follows the MBD approach for WSN application development, but also interconnects a simulated sub-network with a physical sub-network and then allows one to co-simulate them, which is also known as Hardware-In-the-Loop (HIL) simulation.

## 1. Introduction

Advancements in wireless communication modules and Micro-ElectroMechanical systems (MEMs), as well as the huge business potential of widespread low-cost sensing (Internet Of Things—IOT) have motivated the development of small and low power modules equipped with sensors and radios, that are replacing traditional wired sensor systems. These modules can communicate with each other by radio to receive and transmit data and form Wireless Sensor Networks (WSNs).

In the last decade, the landscape of WSN applications has been extending rapidly in many fields such as factory and building automation, environmental monitoring, security systems and a wide variety of other commercial and military areas.

Platforms for WSNs, including processors, sensors, radios, power supplies, operating systems and protocol stacks, are almost as diverse as their application areas, with only a few standards (such as ZigBee [1], 6LoWPAN [2], *etc.*) that mostly address the lower levels of the radio protocol stack, and not the application API. Furthermore, most of the available sensor nodes on the market (such as Mica [3], Tmote Sky [4], MotionBee [5]) only provide a few on-board blinking Light Emitting Diodes (LEDs) for debugging. Although WSNs have experienced great advancements in the last decade, application development in this domain is still quite challenging and time consuming, particularly for application domain specialists who may not be familiar with low-level processor programming and radio control details. There is a lack of tools that provide modeling, Hardware-In-the-Loop (HIL) simulation, and automatic code generation capabilities for multiple platforms from a single high level abstraction. Just like other embedded systems, WSN applications need to be verified functionally before being implemented on the actual platform in order to find as many bugs as possible at a high level, where fixing them has a lower cost. Moreover, the concept of bridging physical nodes with simulated nodes in an integrated framework is still absent from the WSN domain. Bridging the physical world with the virtual one often broadens the possibilities of accelerating and easing embedded system design. This is even more true for WSNs, where generally the developed applications need to be tested and executed in scenarios involving hundreds to thousands of nodes. Since it is hard to manage physical test beds with huge numbers of nodes, the most common solution is to rely on simulation frameworks that allow the developers to create virtual sensor nodes and then provide various abstraction mechanisms (from languages like C to graphical programming environments) to specify the application which will be executed on the nodes. The foremost drawback of this kind of simulation is the absence of direct interfaces with the physical environment, which allow one to include physical details (like the radio or the sensor interfaces) that are hard to model at a high level.

The available functional analysis packages, such as TOSSIM [6] for debugging of TinyOS [7] applications, or OMNeT++ and NS for general-purpose networked applications, fall into two main categories. One is very platform- and OS-specific (such as TOSSIM), while the other includes generic network simulators (such as OMNeT++, NS, *etc.*). Both have significant drawbacks when it comes to complex application development. The first group makes it very expensive to port an application to a different platform (e.g., from TinyOS to a ZigBee [8] compliant platform). The second group still leaves a lot of detailed platform-dependent code to be developed and debugged. Integrated use of a network simulator followed by a platform simulator is the most commonly used path, but it still requires one to port code between a number of environments. Moreover, in case a bug is found at the end, one has to resort to low level debugging, which is extremely time-consuming due to the need to manually maintain the consistency between the various code levels.

**Figure 1.**
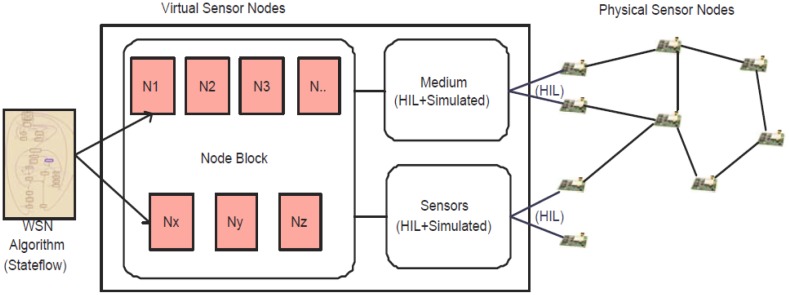
Conceptual view of the hybrid co-simulation framework.

Our contribution is aimed at easing platform-independent WSN application development without compromising the efficiency of the final implementation. We propose a model-based framework built primarily on top of the MathWorks tools [9]. The engineering approach used within the framework follows a development flow consisting of four different steps. In the first phase, called “design phase,” application-level embedded software can be designed and developed using a platform-independent abstraction, namely the Stateflow and Simulink modeling languages, supported by various tools from the MathWorks platform [9]. To remove platform dependency from modeling, we developed a set of generic interfaces (for sending/receiving packets and also for acquiring data from different sensors) and an event-based communication mechanism (based on Stateflow/Simulink signals), which ultimately allows developers to specify applications in Stateflow without knowing in detail the target software and hardware platform. In the second phase, called “simulation,” such models can be configured to execute within a fully simulated environment, including very abstract radio communication models and simulated sensors. In the third phase, called “hybrid simulation,” exploiting the HIL extensions that we developed, it is possible to replace some simulated nodes with physical ones, in order to easily verify hardware-dependent features. In the fourth phase, the same Stateflow/Simulink models can be used to automatically generate code for different WSN platforms (such as TinyOS, or Ember ZigBee), that run on real-world WSN nodes. The hardware dependent features used in the last two phases are the same: for such reason it is also possible to create WSN deployments consisting of any combination of simulated, partially simulated and real WSN nodes. Thus by using this framework, users can build a hybrid network consisting of virtual and real nodes and then simulate it as a whole. A conceptual view of the simulation scenario is sketched in Figure 1.

In this paper, we describe a comprehensive model-based design, simulation and code generation framework for Wireless Sensor Networks by extending work described in [10,11]. In [10], we proposed a single node HIL simulation framework and automatic code generation technique, aimed at providing model-based design capabilities to WSNs. Then in [11] we described a hybrid simulation framework, which mixes virtual nodes with physical nodes. The novel contributions of this paper with respect to [10,11] are in several directions. Firstly, we provide a holistic view of the whole framework by describing both high level application modeling and HIL simulation. In this phase, taking an application of body sensor network as an example, we illustrate in detail how a WSN application can be modeled using our proposed framework. We also provide details of the HIL simulation and automatic code generation process for multiple platforms. Finally, we provide a comprehensive summary of related work.

An earlier version of our proposed framework was described in [12]. That approach suffered from lack of modularity and provided limited support for HIL interactions with the simulation model. In this paper we build upon the foundation described in [12], in order to improve modularity and portability, as well as to provide more complete and orthogonal HIL interfaces. The driving goal of this extension is to provide WSN developers with tools to develop platform-independent WSN application through model-based abstractions, test the implementations through simulation, extend the realism of simulations through HIL (thus introducing hardware dependent features) and finally deploy the code on the nodes, through platform-dependent code-generation. The framework is aimed at improving:

- The time it takes from specification to implementation, by reducing the number of iterations (due to fewer bugs in automatically generated code, and due to a more compact high-level model) and by reducing the manual effort required to obtain the simulation and implementation code.
- The time it takes to obtain derivative designs, again since the model is more abstract than C code and hence more reusable.

Both of these claims are substantiated by a large amount of past work on Model-Based Design (MBD) and Hardware-In-the-Loop (HIL) simulation. *The main contribution of this paper is to show how MBD and HIL can be used for WSN design*, as past work has shown, e.g., for the domain of automotive design. In particular we show that the abstractions (signals, states, transitions, *etc.*) that are useful for MBD of embedded control applications, and that are provided by Simulink and Stateflow, are very close to what is needed for MBD of WSNs. So the only effort required to re-use productively these tools for WSNs is to provide the abstraction layer that we describe in detail in this paper.

The remainder of the paper is organized as follows: in Section 2, a review of related work is presented; in Section 3 the overall architecture of the proposed solution is outlined; in Section 4, we illustrate platform independent modeling using a comprehensive example. We outline the HIL methodology in Section 5 and multi-platform code generation in Section 6. In Section 7, we describe how the framework has been tested in a HIL simulation scenario, including code size comparisons between manual and automated implementation. Finally in Section 8, we present conclusions and future research directions.

## 2. Related Work

**Figure 2.**
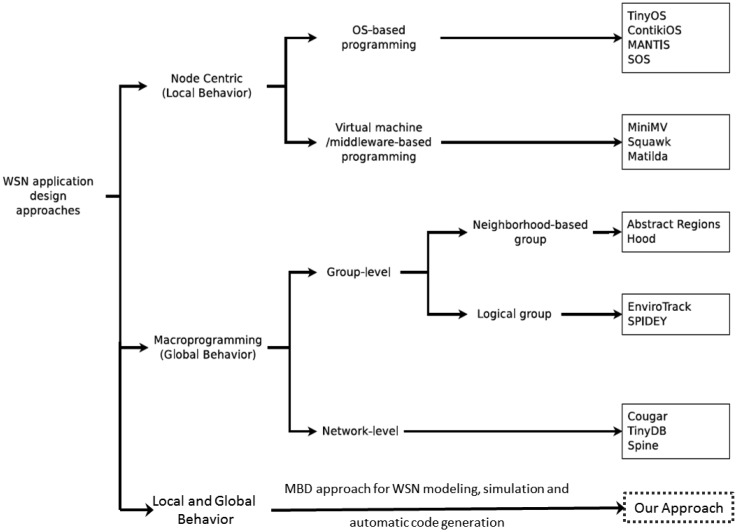
Taxonomy of WSN application design approaches.

In the last decade, much work has been contributed to ease the development of WSN application design using high level abstractions, models and simulation frameworks. According to the established taxonomy in [13,14], they can be classified into two main types (as shown in Figure 2) based on the programming abstractions, namely: node-centric (local behavior) approaches and macro-programming (global behavior) approaches.

In node-centric programming approaches, the global application is decomposed into a set of concurrent local node behaviors, and the programmers have to develop explicitly code running on individual nodes (often called “motes”). The node level programming pattern can be further divided into two groups, namely OS-based programming and virtual machine/middleware-based programming. The OS-based programming pattern offers a flexible control of the hardware resources to the WSN application developers by allowing them to directly interact with the device hardware abstractions. TinyOS (with the associated programming language nesC) [7] is one of the earliest examples in this class and has been widely adopted as the software platform running on numerous WSN devices. There are also other commonly used WSN operating systems utilized by the researchers, such as ContikiOS [15], MANTIS [16], SOS [17], *etc.*

The appearance of light-weight Java virtual machines, e.g., Darjeeling [18] and Squawk [19], allows designers to develop WSN applications using a high level programming language, with slightly reduced application code performance due to the extra resources consumed by the virtual machine. Other techniques based on virtual machine and middleware are also available to relieve the designers from concerns due to the low level aspects, such as a UML-based approach using a virtual machine. Generally, the virtual machine is an execution environment running on top of the operating system.

Macroprogramming approaches introduced a completely different way to develop applications for WSNs. In this kind of approaches, the developers program a flock of motes as a whole, rather than by explicitly writing the software that will run on individual nodes, thus focusing on the overall functionality rather than on implementation and communication details. This can provide flexibility in optimizing the system performance and relieve application developers from dealing with concerns at the mote level. Depending on the scale of the programming entity, the macroprogramming approaches can be classified as group-level (part of WSN) or network-level (entire WSN). Various criteria could be used to define this macroprogramming entity to simplify application development by dealing with data sharing and data aggregation. In macroprogramming, each node belonging to a given group-level programming entity could be considered as a “neighbor” node for other nodes in the same entity. When the group is constructed according to physical closeness (defined by topological distance, *i.e.*, number of communication hops, or geographical distance), it can be called a “neighborhood based group”, such as in Abstract Regions [20,21]. On the other hand, if nodes are grouped based on some high level logical properties (e.g., same node type or same physical quantity to be measured), then the group is called a “logical group”, as in EnviroTrack [22]. The APIs exposed by a programming group (both neighborhood-based group and logical) can hide the communication inside that group, thus making it easier for the developers to design a collaborative algorithm and analyze its performance. When the group is extended to enclose the entire WSN, then it becomes a network-level programming entity. Solutions such as COUGAR [23], TinyDB [24] and Spine [25] employ this kind of approach. They leverage distributed database techniques and extensible processing and query mechanisms to abstract the underlying network as an entity. Within these solutions, a task is described in high-level languages, injected in the network and transformed into low-level procedures running on each individual node. However, macroprogramming partially reduces the possibility to obtain a fine-grained control over application logic due to the limited expressiveness of high-level task description languages.

In contrast to the node-centric and macroprogramming approaches, in this paper we provide rich abstractions that allow one to model application behavior still at the node level, yet in a platform-independent and thus more abstract fashion that classical node-centric approaches. Moreover, users can exploit HIL simulation to connect with real world sensors and networks to perform hybrid network analysis. Finally, the behavior that has been modeled and refined during the simulation phase can be used to generate application code for WSN platforms such TinyOS, Mantis, a commercial ZigBee stack and others. To the best of our knowledge, there is no framework available that can provide these capabilities for WSN application development.

Simulation is a de-facto standard first-step in the implementation of a WSN-based solution. However, it is not completely reliable, for many different reasons. On one hand, the degree of realism of the simulation depends on the complexity of the underlying simulation model, which is often based on unrealistic over-simplified assumptions (e.g., regarding the communication models). On the other hand, code executed by the simulator is typically different from the software running on actual WSN nodes, mainly because of differences in the HW platform, which introduces discrepancies regarding simulation timing, concurrency and performance. Finally, even in cases where the simulation code is very similar to the actual code (such as in [6]), the simulation necessarily behaves differently from actual code regarding access to platform-dependent components, such as radio transceivers or sensors. For such reasons, WSN developers are usually forced to engage in time-consuming debug sessions when they move from the simulation to the actual deployment phase. Our hybrid simulation approach, mixing real and simulated objects through HIL interfaces, helps reducing efforts in this intermediate phase.

Hybrid Hardware-In-the-Loop simulation is a consolidated approach in several embedded system application domains, such as industrial automation, automotive, and so on [26–28]. In hybrid simulation, some parts of the system are simulated and other parts are real-world components and sub-systems, integrated through HIL. A co-simulation framework usually is in charge of controlling the interactions between the simulated and the real world. Despite complexity and technical difficulties, hybrid simulation is valuable because it can couple the realism of actual deployment with the flexibility of simulation, in particular keeping the former small scale, while the latter deals with large numbers of nodes. Hybrid simulation has already been used in WSNs, for both testing and debugging purposes. In [29] hybrid simulation is used specifically for testing embedded software for TinyOS-based applications, using a wireless-based control channel. In EmStar/EmTOS [30], hybrid simulation (called “emulation mode”) is used as a means to enhance simulation with real radio channels. In [31], hybrid simulation is used to extend TOSSIM capabilities, employing a time-freeze strategy to keep the simulation running at pace with the real world. In [32] the same approach is used, but with the opposite purpose, *i.e.*, to “augment” a real network with a set of simulated nodes.

In [33], the authors propose a toolset to support application development for a novel FPGA-based sensor node platform. In this hardware/software co-design approach, a flexible hardware/software boundary can be specified by the user (based on the mechanism of “late binding” of application components) in order to optimize the overall performance. However, the approach is specific to the proposed experimental platform and still requires the application developers to master knowledge about operating systems, communication protocol stacks and integrated circuit design.

In [34], a comprehensive extensible meta-data specification and the corresponding meta-model are proposed which support the description of components and configuration of WSNs. Based on this approach, various types of networks can be described at different levels of detail in a service-oriented style, which still requires from the application developers a lot of knowledge of network protocols and platform specifications.

In [35], the authors propose a model-based optimization framework dedicated to wireless body sensor networks (WBSN). Based on the research and analysis of the most energy-demanding components in WBSN applications, a multi-objective optimization algorithm is proposed in to find the optimal tradeoffs available in the design space. However, they do not discuss how to manage network simulations and HIL simulation using MBD features.

In [36], the authors introduce SenseWeaver, a SysWeaver plug-in that supports model-based design of wireless control applications. They propose a top-down model-based design approach to create wireless sensor-actuator networks. The approach manages complexity and enables the automatic integration of multiple applications. The component libraries allow code to be cleanly integrated and reused across different applications, thus reducing the amount of hand written code and the development time.

Even though as described above research has made significant progress towards delivering an integrated tool suite for WSN application modeling, simulation and automatic code generation, many aspects still require more work. Indeed, many of the above mentioned tools and methodologies are not suited for describing complex designs or lack some modeling aspects which are essential to create a fully integrated co-design environment which can actually be used in an industrial setting. As a typical example we can cite approaches based on SystemC modeling [37], which are simple to use and powerful, but require a virtual machine on the node to execute the code, and hence are not optimal for low power and high performance applications.

Similarly, while UML-based tools are in widespread use to model and document enterprise software, their adoption in the embedded space is still limited. For this reason, even though we could have started our MBD approach from UML or SysML, in this work we propose using a widely known and widely used industrial language, namely Stateflow/Simulink, as the starting point of our work. Please note that most ideas behind our approach are fully applicable to a UML-based flow.

## 3. Hybrid Simulation Framework

**Figure 3.**
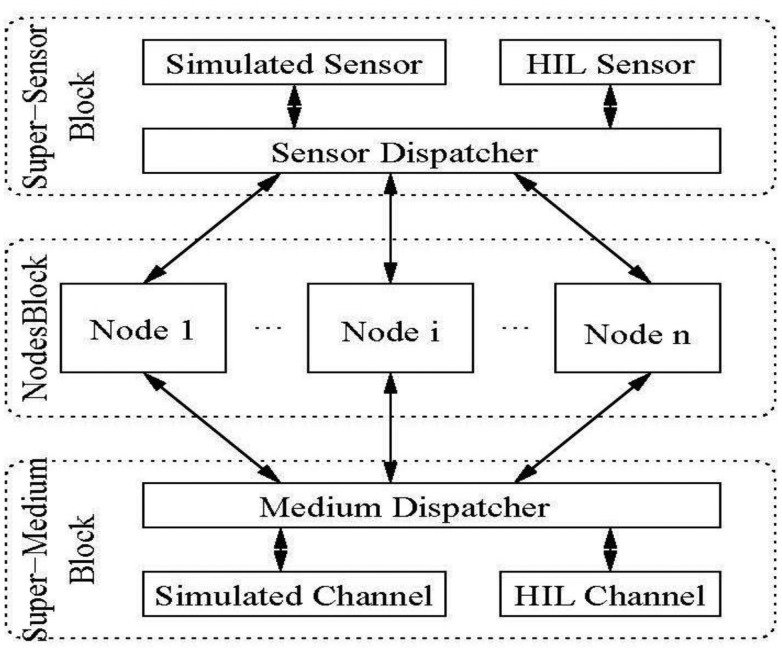
Architecture of the hybrid simulation framework.

The architecture of the framework, implemented using Simulink/Stateflow [10], is depicted in Figure 3. A brief description of the functionality of each component in the framework is given below. The top level contains three “super” blocks which respectively hide and abstract: (1) sensing/actuation; (2) application functionality and (3) communication.

(1) **Nodes Block**. This is a container block which abstracts application functionality as a set of cooperating processes (*i.e.*, Stateflow StateCharts or code running on physical nodes). It contains a separate instance for each node included in the scenario. Node objects can be fully simulated, partially simulated or independent.
  - Fully simulated nodes are completely handled inside the simulator, as Stateflow Statechart instances.
  - Partially simulated nodes are modeled inside the simulation framework but are able to access HIL components (such as sensors, or transceivers) which reside on physical devices.
  - Independent nodes are real physical nodes, running code generated from Stateflow, which interact with fully or partially simulated nodes only through the radio channel and a framework-provided interface with the Super-Medium block.
(2) **Super-Medium Block**. This is a component devoted to managing all radio communications within the scenario. In particular, it is able to dispatch packets within the simulated world and also between the simulated and the real world, via one or more physical nodes connected to the simulation host, as described below.
(3) **Super-Sensor Block.** This is a component devoted to managing all sensing aspects within the scenario. It is able to provide simulated sensor readings and means to access actual sensors through HIL interfaces.

In the following subsections, the Nodes, Super-Medium, and Super-Sensor blocks are illustrated in more detail.

### 3.1. Nodes Block

**Figure 4.**
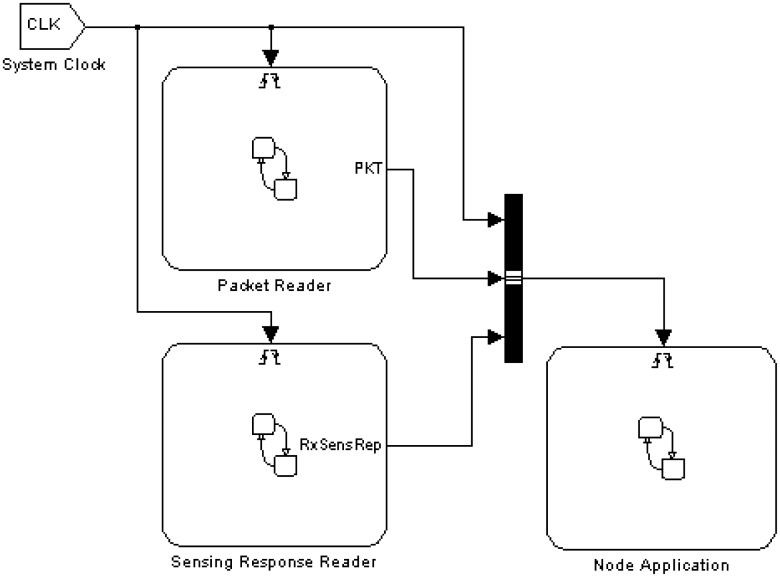
Node instance structure.

The Nodes Block is an abstraction that contains nodes which will interact with each other in the application scenario. Each node is modeled as a separate instance, structured as shown in Figure 4.

**Figure 5.**
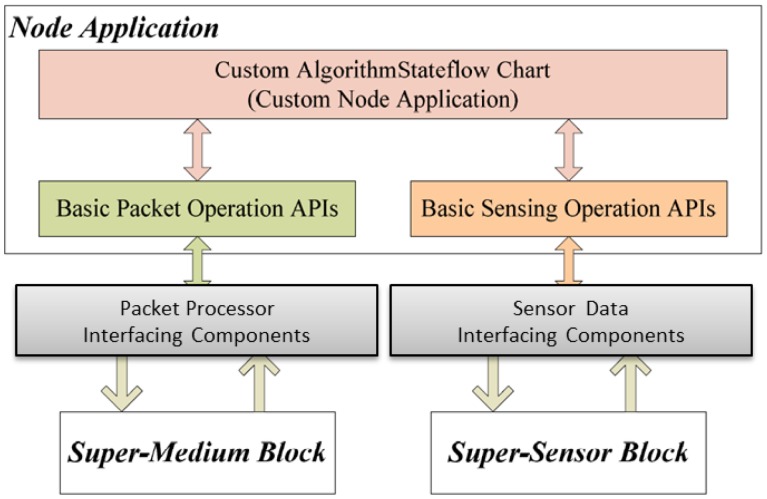
Interface between node application and other simulation components.

The Node Application is a block representing the platform-independent functional model of the WSN application running on each node. The Node Application can interact with the rest of the world through the Packet Reader and Sensing Response Reader, providing a direct connection with the Super-Medium and the Super-Sensor blocks respectively. The Packet Reader behaves as a radio receiver: upon detection of a radio packet, it generates an event called PKT for the Node Application instance, making the payload of the received packet available to it (we will see below how packet transmission is modeled). Similarly, the RxSensRep event is used by the Sensing Response Reader to notify the application of the availability of a sensor reading. A System Clock synchronous signal, finally, provides global timing information to the node components. The “logical” interface to communicate between the node application and other framework components (sensors and medium) is depicted by the block diagram in Figure 5. The node application can communicate with both the “Super-Medium Block” and “Super-Sensor Block” using platform independent APIs to get and put radio packets and sensor data, which can be used transparently in simulation, HIL and code generation modes. The users are able to develop the node application independent of the simulation setup and the node implementation platform, using these APIs and events (e.g., PKT and CLK). These API calls will be translated to the target platform (in the form of OS calls, driver calls, *etc.*) during the code generation phase.

Although each node instance is structured according to the same scheme, its actual behavior within the simulation depends on its type. Five different node types are supported in the framework: SIM, HIL_SEN, HIL_RF, HIL_FULL and REAL. The types differ on how they interact with the Super-Medium and the Super-Sensor, as described in the following. Note that the application model is totally unaware and independent of the node type, since the framework handles these aspects transparently.

- SIM (SIMulated): this fully simulated node uses both virtual sensors and a simulated radio transceiver.
- HIL_SEN (Hardware-In-Loop SENsor): this partially simulated node uses the simulated radio transceiver, but collects sensor data from actual sensing devices through the Super-Sensor.
- HIL_RF (Hardware-In-Loop Radio): this partially simulated node collects data from virtual sensors, but uses the actual transceiver through the Super-Medium for sending and receiving packets.
- HIL_FULL (Hardware-In-Loop FULL): this partially simulated node executes the application code within the simulation environment, but uses both actual sensors and the actual transceiver.
- REAL: this fully independent node, existing only in the physical world, can have active radio communication with other types of nodes, thanks to the Super-Medium, which uses a stub physical node to communicate with it. The application running on it could be coded manually (*i.e.*, using platform-dependent programming languages and operating system, such as TinyOS), or it can execute the automatically generated code from the Stateflow model of the Node Application.

During the initial scenario definition phase, users can instantiate any number and combination of the aforementioned node types. For partially simulated nodes, the WSN developer can specify the association between any WSN node instance and HIL interfaces in the Super-Medium and Super-Sensor blocks. This relationship associates partially simulated nodes with actual HIL radio transceivers or sensors. Note that multiple HIL nodes can be associated with the same physical device, but this procedure must be handled carefully, because it can generate inconsistencies (e.g., it is not possible to send two radio packets at the same time using the same radio physical radio transceiver). The Super-Medium node currently resolves the resource contention by dropping the collided communication (which is realistic if the HIL nodes are meant to be in radio contact with each other).

The main idea behind our framework is to provide a simple, yet modular and flexible approach to extend high-level, WSN application simulation with HIL, letting the WSN developer specify the hybrid simulation configuration on-the-fly.

### 3.2. Super-Medium Block

The Super-Medium block manages the exchange of packets within the framework, including any combination of fully simulated, partially simulated, and real nodes. It works by performing “read” and “write” operations over tuples of incoming and outgoing buffers, one tuple per node in the scenario. Packets are exchanged using intermediate buffers which work like placeholders between different components. Depending on simulation parameters such as: (1) the type of the transmitting node; (2) the type of the receiving node; (3) the type of link between the two components; and (4) the simulation radio model configured by the user, a different kind of packet dispatching is performed. For instance, a packet directed to a REAL node might be transferred directly to an actual device through the HIL interface, while a packet exchanged among two SIM nodes is first processed by the simulated radio channel model and then, in case of success (no packet error), simply transferred between the two virtual nodes.

**Figure 6.**
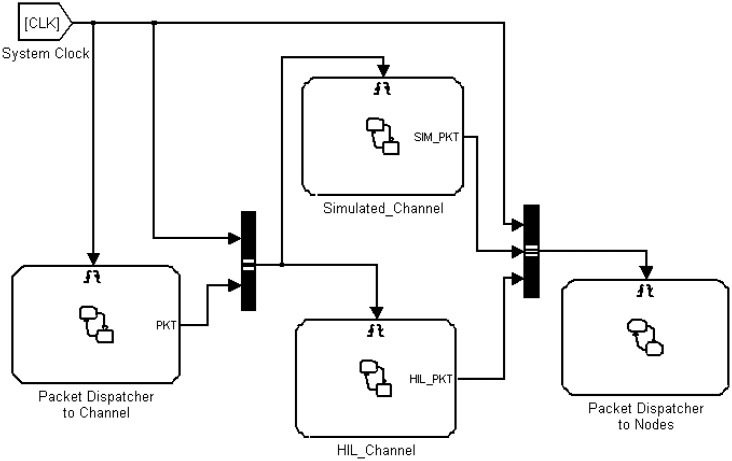
Structure of the Super-Medium Block.

A snapshot of the Super-Medium Simulink block is presented in Figure 6. It is composed of four sub-blocks, namely the Simulated Channel and the HIL Channel, plus two blocks used to handle communication from and towards nodes. The role of such blocks is to collect all packets sent by node instances, process them either via simulated radio models (Simulated Channel) or using actual radio channels (HIL channel) and then notify the packet reception event to all nodes that receive packets.

#### 3.2.1. Simulated Channel

Currently the Simulated Channel uses a simple implementation based on a link-quality matrix specified in the configuration phase, containing packet loss probability among any couple of nodes. Such implementation can be replaced by other simpler or more complex radio model implementations. The current implementation does not take into account possible interference among transmitted packets, but this can be easily added into the model.

#### 3.2.2. HIL Channel

The HIL Channel handles radio communication from and to actual radio nodes. Its role is to intercept and handle packets to be broadcast on the physical radio interface to an HIL_RF, HIL_FULL, or REAL node. The HIL Channel controls, through serial cables, one or more physical nodes used as radio interfaces.

A software stub running on the physical devices is responsible for dispatching radio packets from the physical interface towards the framework and vice-versa.

When the HIL_Channel component receives a packet through the HIL interface, it is able to decode the packet header and dispatch the packet to the correct destination node (or nodes) according to the association between the node ID and the HIL interface.

### 3.3. Super-Sensor Block

Since sensor interactions are usually local to a node, its design is much simpler than the Super-Medium, though they share some similarities. Each node instance is provided with an incoming sensor data buffer. When a new sensor reading is available, the Node Application is notified.

As described before, the Super-Sensor node is able to receive sensing requests from all node instances. Upon reception of a request, the Super-Sensor generates a sensing value in different ways, according to the node type and the request type. In case of nodes with simulated sensors (SIM, HIL_RF), the sensor reading is generated according to a simulated model (e.g., a sample data file) defined in the initial configuration phase. In case of nodes with HIL sensors (HIL_SEN or HIL_FULL), a request is routed to a specific HIL interface connected to a physical node (which can be the same as the one used by the Super-Medium for the radio, or a different one).

**Figure 7.**
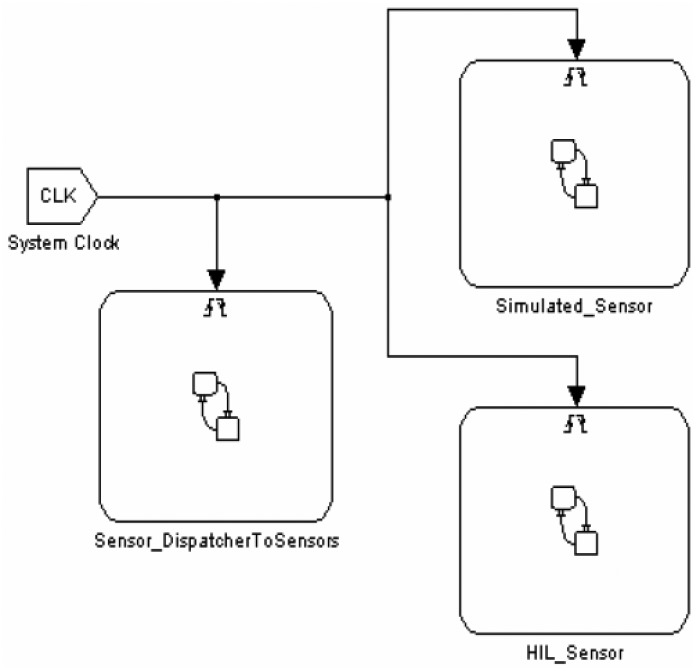
Structure of the Super-Sensor Block.

A snapshot of the Super-Sensor Simulink block is depicted in Figure 7. The Super-Sensor is composed of three sub-blocks: Simulated Sensor, HIL Sensor and a dispatcher handling data exchange between sensors and the node application.

## 4. Platform Independent Algorithm Modeling: An Application Case Study

In this section, we provide a detailed example of how we used Stateflow/Simulink to model a non-trivial WSN algorithm (which could belong to the application or middleware layers) inside the Node Application block (shown in Figure 4). Faithful to the model-based design approach, the application developer uses Stateflow constructs (such as states, transition diagrams, events, function calls *etc.*) which are independent of the specific platform and programming languages (such as TinyOS or implementations of the ZigBee stack) which will be used on the physical nodes. Interactions with the radio channel and the sensors are performed via function calls and events which are part of the framework. As mentioned above, each node can interact with virtual (simulated) or physical (connected by HIL interface) radio and sensors. We provide abstract interfaces for accessing transparently both the virtual and the physical components (radio and sensors), which decouple high-level application modeling from simulation scenario configuration. Hence we can use this platform-independent application model for automatic target code generation as well as for simulation.

To illustrate our flow, we use a realistic application that consists of a virtual machine oriented to data processing in Body Sensor Networks which is very similar to Signal Processing in Node Environment (SPINE) [25]. A SPINE node is able to perform dynamically configurable signal processing computations (such as max, min, median, *etc.*) based on collected data sets from sensors. The parameters of these computations (called “features”) such as sampling rate, computation window, shift of data set at each new sample, *etc.* can be tuned and the features can be activated or deactivated depending on the application demands. One of the prominent applications of SPINE is to detect body movements, hence a three axis accelerometer is used as an example in our SPINE implementation. Our model of SPINE in Stateflow contains three parallel state machines and 23 Stateflow functions, which implement the following main functionalities:

- **Scheduler:** It manages the active tasks of the system. A task can be configured to perform the following actions reading a specific sensor at a specified interval (sampling rate), storing the sensor readings into a circular buffer and then calculating features (for example max, median, *etc*.) using the stored data based on a given window/shift value. After computing a feature, it sends the result to the base station via the radio.
- **Circular buffer:** It is used to store sensor readings in so-called segments. Each segment is used to store sensor readings from a specific sensor and channel (for example the x, y or z axis values of the accelerometer).
- **Packet processor:** It decodes incoming configuration packets that are sent by the base station to configure the activities of the sensor node.
- **Features:** Our SPINE model can perform some computations (such as max, min, median, *etc*.) on the data sets stored in circular buffers.

**Figure 8.**
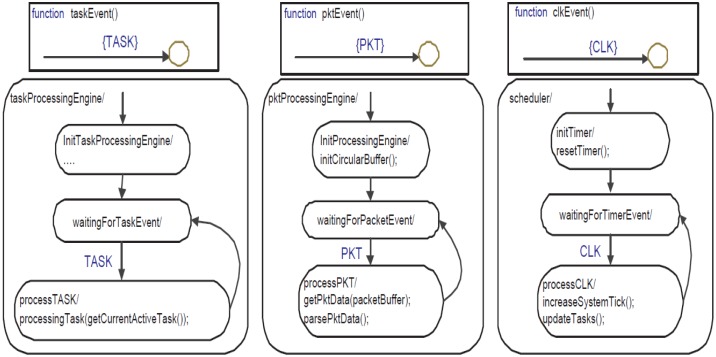
Three parallel state machines of SPINE.

**Figure 9.**
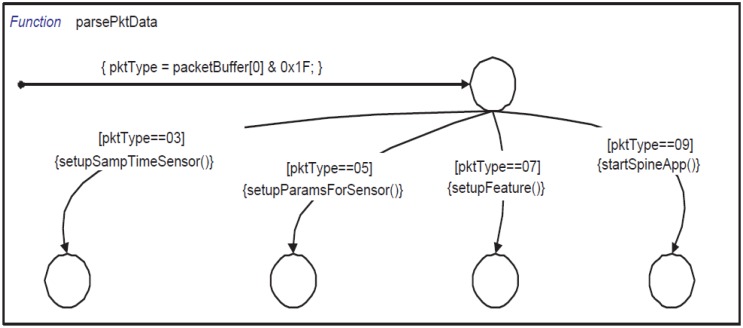
Stateflow function parsePktData—used to parse configuration packets sent by the base station.

The three parallel state machines (taskProcessingEngine, pktProcessingEngine and scheduler) process external events like CLK, PKT, RxSensRep and internal ones like TASK. A snapshot of these state machines is shown in Figure 8. Initially, these three state machines start in parallel and after initialization wait for incoming events to be processed immediately. The base station activates tasks by sending several configuration packets. When pktProcessingEngine receives a PKT event (generated by serial_port_packet_reader), it immediately calls getPktData which copies the packet payload into a local buffer (packetBuffer). Afterwards, it calls parsePktData (shown in Figure 9) which processes different types of configuration packets.

In our SPINE implementation, we process four different types of packets that are used to configure the basic SPINE virtual machine:

- **Packet Type 3:** It contains data that are used to configure the sampling time of individual sensors.
- **Packet Type 5:** It contains general information to define features such as window (number of samples needed to compute a feature for the first time) and shift (number of samples needed to compute a feature after the first time).
- **Packet Type 7:** It contains data about the features (max, mean, *etc*.) that need to be activated or deactivated. It also contains information on which sensor and channel (x-axis, y-axis, *etc*.) must be used for these features.
- **Packet Type 9:** This packet is used to start/stop all the tasks managed by the SPINE engine.

For example, The Stateflow function setupSampTimeSensor parses packet type 3. It extracts the sampling scale (millisecond, second or minute) from the payload data. Afterwards, it extracts the sensor code and sampling coefficients and then calculates the sampling time for that sensor and inserts it in the active sensor list. Similarly, setupParamsForSensor extracts the window and shift value for each active sensor, and setupFeature selects the features that need to be calculated. The setupFeature function also assigns which portion of the circular buffer will be used for each specific feature. Finally, the startSpineApp function adds all the features as active tasks in the scheduler.

**Figure 10.**
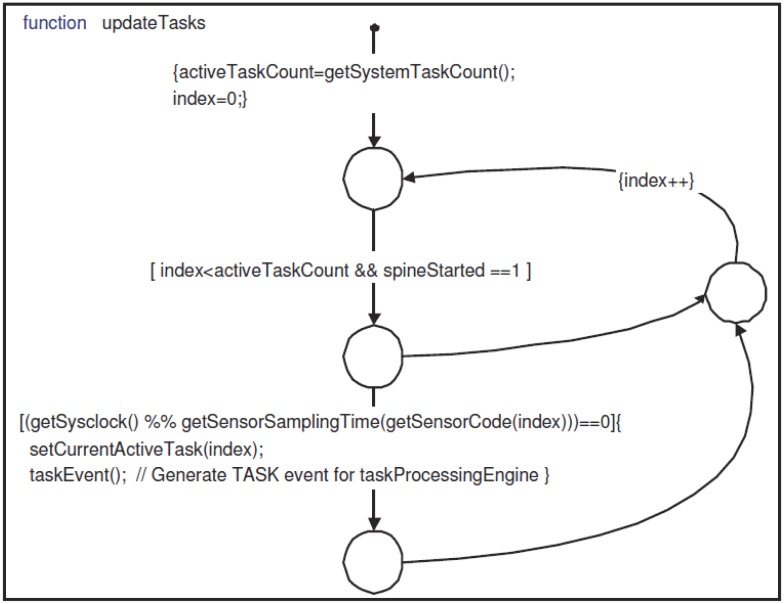
Stateflow function updateTasks—schedules tasks for execution.

**Figure 11.**
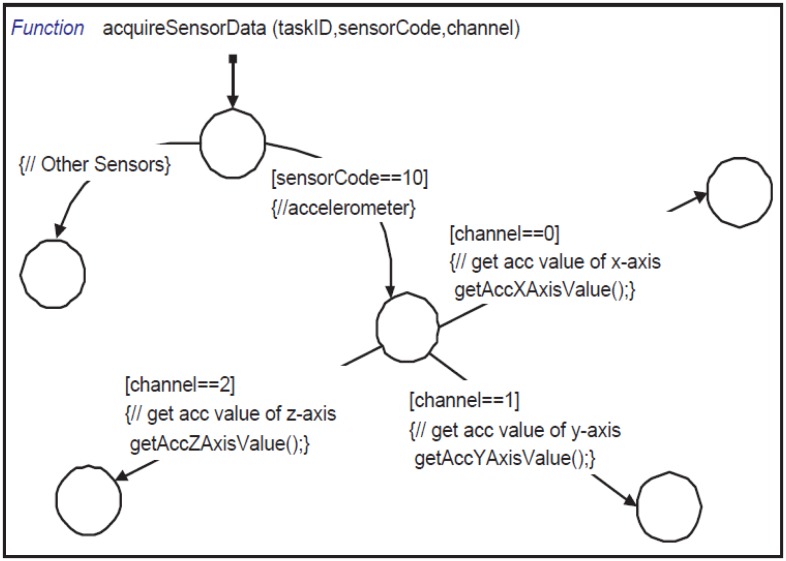
Stateflow function acquireSensorData—used to read sensor data from different sensors.

The scheduler, after initialization, waits for the CLK event. At each CLK event, it increments the system timer count and then calls updateTasks (shown in Figure 10). Inside updateTasks, it checks the sampling time for each active task. When a task needs to acquire data from a sensor, it is activated by generating a TASK event for the taskProcessingEngine. The taskProcessingEngine waits for TASK events to process the active task by calling the processingTask function. Inside processingTask, it decides whether the type of the task is an alarm or a feature. If it is a feature, it calls acquireSensorData (shown in Figure 11) to collect data from sensors. The acquireSensorData function uses framework-provided calls to read simulated or physical sensor data.

**Figure 12.**
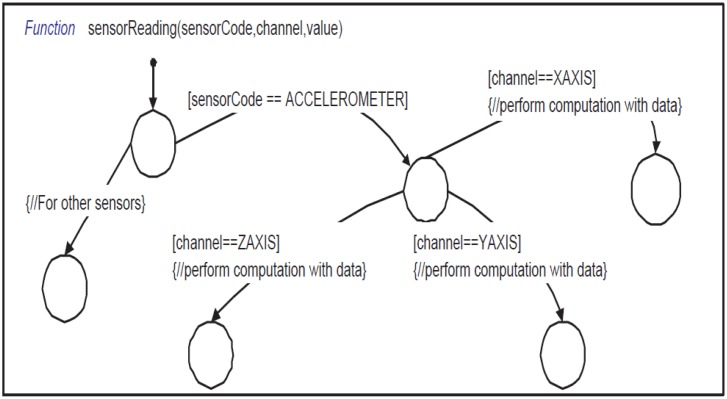
Stateflow function sensorReading—used to return sensor reading to the Stateflow application model.

These calls are processed by the Super-Sensor block. Then Sensing Response Reader (shown in Figure 4) generates an RxSensResp event when sensor data are available. Please note that we use an asynchronous (split-phase non-blocking) request/response mechanism for efficiency, but a synchronous (blocking) communication with the sensors can easily be supported by extending our API. The RxSensResp event generated by the *Sensing Response Reader* triggers the sensorReading function (shown in Figure 12) which stores the acquired sensor data into the specified segment of the circular buffer. When adequate data sets are available in the circular buffer, the state machine computes the specified feature (max, mean, *etc*.) of each active task. It then constructs a payload with the feature result and sends it to the Super-Medium by using the sendPacket Stateflow function. After receiving the payload, the Super-Medium broadcasts it according to the network setup.

**Figure 13.**
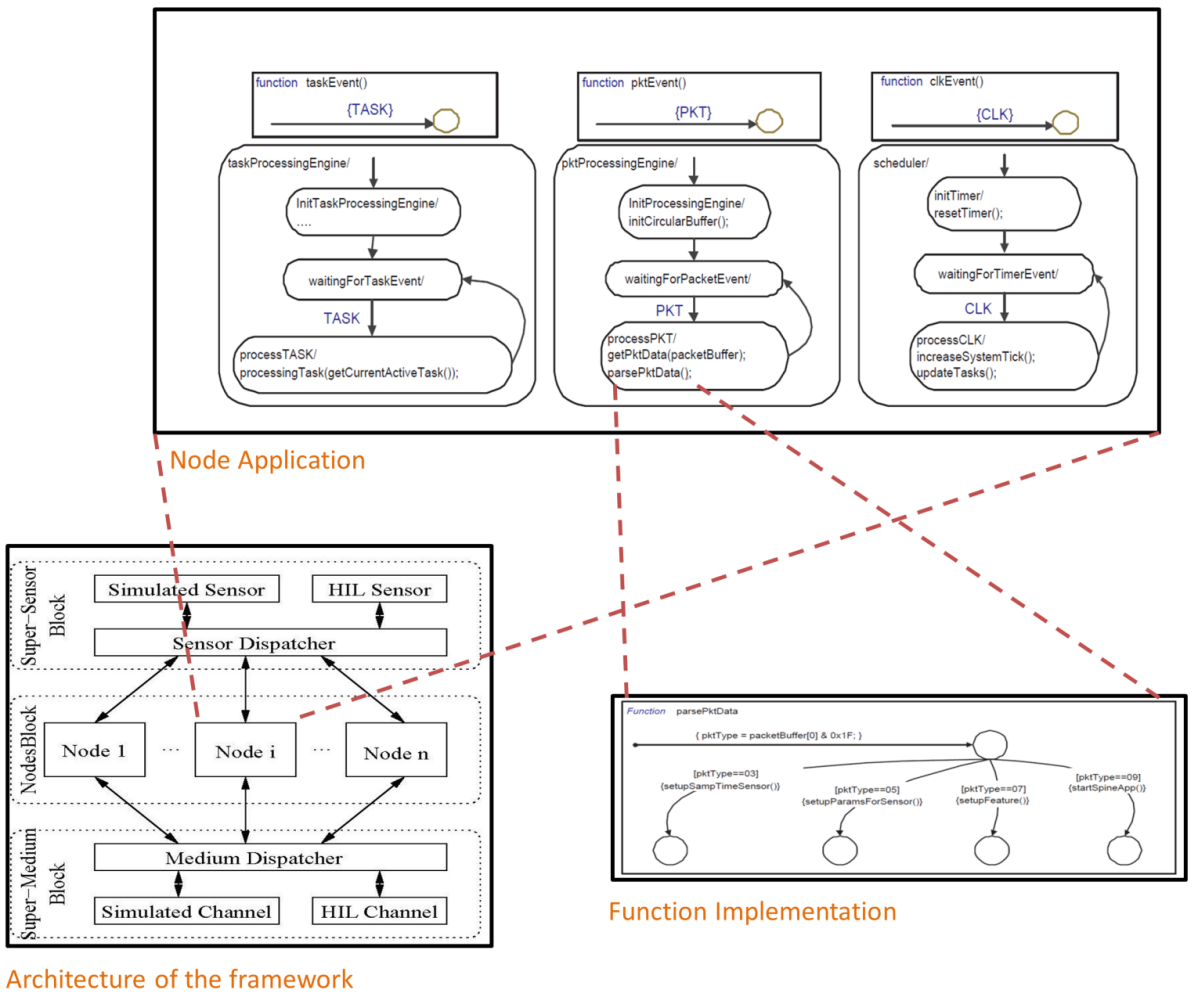
Relationship between simulation framework and its instances for application development.

Figure 13 depicts more in detail the relationship between the simulation framework, including the simulated node instances, the StateFlow model for the application, and finally the functions that the StateChart calls to perform computations. Each level of the modeling hierarchy can be navigated up and down using the features of the StateFlow model editor.

The Stateflow-based or Simulink-based WSN application modeling approach could be used to model applications of any sort. In [38], for example, we modeled a data aggregation algorithm for a cluster-based sensor network using Stateflow. Similarly, WSN applications such as distributed algorithms for dynamic task assignment [39], for resource management using collective intelligence [40] and others could be easily modeled using our proposed approach. In this example, we used Stateflow since it is appropriate for a reactive application like SPINE. Simulink blocks could be used to model more data-intensive applications as well.

## 5. Hardware in the Loop Simulation

The HIL interface is used by both the Super-Sensor and Super-Medium blocks to access physical sensors and radios connected to the simulation host e.g., via serial ports. HIL support includes mainly two entities: the stub code executed on the physical node containing the sensors or radio, and the Simulink block (inside the Super-Sensor and Super-Medium blocks) used for accessing this stub via a serial (e.g., RS-232 or usb) cable. The stub contains basic platform-dependent code to join or interact with a ZigBee or TinyOS network, without any application-specific part. When the stub receives a packet from the network, it stores the packet locally and at the next request from the Super-Medium block, it transfers the packet payload over the serial cable. In the same way, when the stub receives a packet payload from the Super-Medium block, it constructs the actual packet and transmits it to the network. The Super-Sensor block interacts in a similar way with the stub for reading sensors. In the following, we will use as an example a three-axis accelerometer included in a node from STMicroelectronics [5]. We will use it for HIL simulation with two supported platforms that are very different from each other: TinyOS and the ZigBee-compliant Ember stack. Note that the same methodology can be used to extend the framework to any number and kind of WSN platforms.

As mentioned above, each stub contains device drivers and other basic software to support the underlying platform. In addition, it includes a simple serial protocol implementation that is used to communicate with the Super-Sensor and Super-Medium. This protocol is used for reading data from sensors and also for sending or receiving packets to/from the network respectively. To maintain smooth communication, the serial protocol is implemented using several transactions, all initiated by the master (Super-Sensor and Super-Medium). This is similar to the USB protocol, but can be implemented on top of any kind of connection (e.g., USB, RS-232, *etc*.). The protocol uses the following commands to interact with the blocks:

- SENDPKT: When the stub receives this command at the starting of a new transaction, it knows that it is going to receive a packet payload from the Super-Medium. The next byte should be the length of the payload followed by consecutive bytes of the payload. After receiving a complete packet, it constructs the physical packet and broadcasts it to the network.
- GETPKTCNT: After receiving this command from the Super-Medium, the stub sends a byte which contains the number of currently stored received packets.
- GETPKT: After receiving this command, the stub transfers the received packet to Super-Medium. Afterwards, it removes the packet payload from the queue. When the stub receives a packet from the network, the stub stores it in the local queue and then transfers it to the Super-Medium by using transactions GETPKTCNT and GETPKT.
- GETACCELXAXIS: After receiving this command from the Super-Sensor, the stub calls either LIS3LgetX.get (on TinyOS) or getAccXAxisValue (on Ember ZigBee) to read the X axis value of the three axis accelerometer. In TinyOS, this is an async command whose result is returned by calling the LIS3LgetX.getDone event handler. Inside this event handler, the stub transfers two consecutive bytes of the result to the Super-Sensor block. In Ember Zigbee, the getAccXAxisValue function is executed in a blocking fashion and directly returns two bytes of the result, which are then sent to the Super-Sensor block. Note how this interaction, modeled as an asynchronous call (AcquireSensorData) followed by an event (RxSensResp)on the Stateflow side, is implemented (1) an asynchronous split-phase call (LIS3LgetX.get/LIS3LgetX.getDone) on a TinyOS node and (2) as a synchronous call which generates the callback event on a ZigBee node. The application programmer can thus ignore the tasking model and other architecture details of the target platform. Our HIL and code generation framework takes care of generating the most efficient implementation.
- GETACCELYAXIS and GETACCELZAXIS perform the same for the Y and Z axis.

## 6. Multi-Platform Code Generation

**Figure 14.**
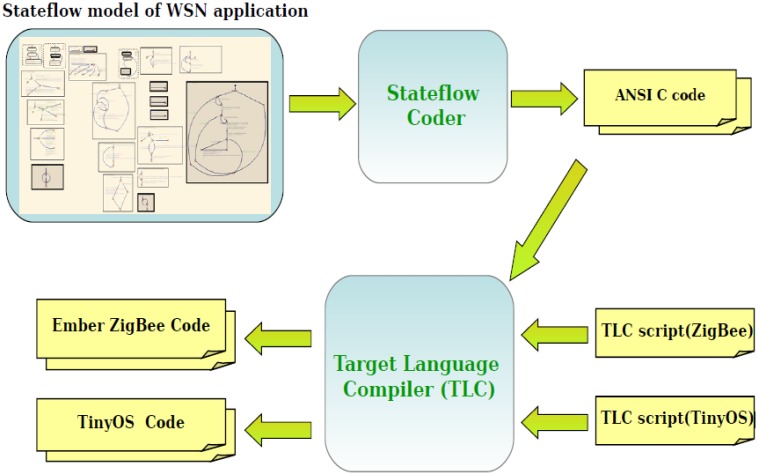
Multi-platform code generation from Stateflow.

As mentioned in the previous sections, the WSN algorithm is modeled inside the Node Application block (shown in Figure 4). The whole flow to generate platform specific code for this block is depicted in Figure 14. For each target platform (TinyOS, Mantis, the Ember ZigBee stack implementation) we must create a custom Stateflow target for ANSI C code generation which will automatically generate application code for that platform [41].

The computational bodies of the functions called by the task handler in the Ember ZigBee stack, as well as those of the TinyOS tasks and event handlers, are essentially written in C. So the code generated from Stateflow coder can be directly ported to Ember and TinyOS with almost no modification. Similar to the stubs for the HIL interface, we developed platform specific code for the TinyOS and Ember platforms that will interact with the ANSI C code generated from the Stateflow model of the Node Application block. This code also contains platform specific implementation of all framework-defined calls (sendPacket, getPacketPayload, getAccXAxisValue, *etc*.) and event processing functions for CLK, PKT, RxSensRep, *etc*. In order to pass an event to the Application block, we just call the corresponding event processing function from the framework-provided base code, which in turn calls the generated code of the Node Application block with the corresponding event.

In other words, our framework provides a platform for modeling WSN applications in Stateflow that is independent of the underlying OS and programming language. The functions and events provided by that platform, e.g., the sendPacket and getPacketPayload functions and the CLK and PKT event are translated into the underlying OS platform by a very lightweight and efficient application-independent framework layer that we provide, and that is called “base code” in the following.

A skeleton of the TinyOS specific base code is shown in Example 1. To send the CLK event to the application, we use a periodic timer (Timer0 in the example). Since this file will be automatically generated by TLC scripts [42], we can decide the duration of the CLK pulse at code generation time. On each occurrence of the periodic timer, the clkEvent (event processing function) will be called, which will pass this event to the Node Application platform-independent code. In TinyOS, the driver implementation for the STMicroelectronics accelerometer is done asynchronously, as mentioned above.

**Table:** 

**Example 1: Skeleton of platform base code in TinyOS**
module wsn_applicationM
{ …}
implementation
{
….
event void Boot.booted(){
….
call Timer0.startPeriodic(10); //CLK pulse in every 10ms
}
event void Timer0.fired() {
clkEvent(); //Up Call: Sending CLK event to the wsn_application
}
event void LIS3LgetX.getDone(uint16_t xAxisValue, error_t success){
//Up Call: Returning X-axis value of the ACCELEROMETER to the wsn_application sensorReading(accelerometer,xaixs,xAxisValue);
return;
// Down Call: To read the X-axis value of accelerometer void getAccXAxisValue() @C() @spontaneous() {
call LIS3LgetX.get ();
}
// Down Call: Construct packet with payload and broadcast it. void sendPacket(char* payload) @C() @spontaneous(){
…
}
event message_t* Receive.receive(message_t* msg, void* payload, uint8_t len) {
// Store packet payload locally
pktEvent(); // Up Call: Send PKT event to wsn_application
….
}
// Down Call: To get the payload of the packet void getPktData(char* payload) @C() @spontaneous() {…}
}

The Node Application code calls getAccXAxisValue (down-call) to read the x-axis value of the accelerometer. Inside this function we call LIS3LgetX.get, which returns immediately due to the split-phase semantics of TinyOS. Afterwards, when the value is read from the accelerometer, the driver calls LIS3LgetX.getDone with the sensor value. Inside this function, we call sensorReading (up-call with values) which provides the sensor reading to the Node Application. On the other hand, synchronous reading (for the Ember Zigbee stack) is fairly easy because we can call sensorReading inside the getAccXAxisValue call. We followed the same techniques to glue the base code with platform independent code in all the platforms that we support.

### Platform Specific Code Generation by TLC Scripts

The approach that we explained in this section is automated by appropriate TLC scripts. The application code for the TinyOS and Ember ZigBee platforms looks very different, so the TLC script performs the following tasks to generate platform specific code:

- Copy into the target files the platform-specific application-independent base code, which includes (1) a type conversion header file that converts standard C types to platform specific types and (2) platform specific implementations of the library functions provided by our framework.
- Generate platform specific application files by taking different sections (such as includes, defines,functions, *etc.*) from the C code generated from Stateflow, and inserting them into the appropriate location of the platform-specific files.
- Generate make or configuration files for the platform.

## 7. Experimental Results and Code Size Comparison

In order to illustrate how to design, simulate and test the WSN application based on this framework, we use first a small example, and then provide some more results for the more realistic SPINE application. The small example implements a token ring network in which each wireless sensor node:

- awaits for the token (transmitted via the radio channel),
- collects several acceleration sensing values, and
- transmits the token to the following node.

Tokens are passed according to the node ID. When the node receives the token, it collects three acceleration values on X-axis, four acceleration values on Y-axis and five acceleration values on Z-axis. Then the current active node sends out the token and enters into sleep mode until it is woken up by receiving the token again.

After designing the WSN application, the user should set the configuration of the nodes and of the network. A Matlab file is used to perform this customization: the user can easily modify it by hand, but we are also working towards the implementation of a graphical user interface to further simplify this operation. The execution of this script automatically generates the SuperMedium and SuperSensor blocks, and sets up the environment for the simulation. In case of HIL nodes, this operation configures also the hardware interfaces to allow the communication among simulated and physical nodes. The application was tested with a different number of nodes, to give an idea of the scalability of our approach. Table 1 shows the simulation time for different network sizes: the first column contains the number of nodes in the network. For each size the overall execution time is reported, together with the average execution time for the different node types included. After the model has been developed and simulated, we generated the application code automatically for both the TinyOS and the Ember ZigBee platform. Firstly, by using Stateflow Coder, we generated ANSI C code for the application. Then by executing TLC scripts we added platform dependent code. Table 2 reports the size of the automatically generated code for both platforms. In this table we also noted the size of the platform base code (without any application code), to give an idea about the relative importance of application-specific and application-independent code in a very small example application.

We also generated code for SPINE, which is a more realistic example of WSN application, for both TinyOS and the Ember ZigBee stack, as shown in Table 3. To estimate the code size increase due to automation, we also implemented manually the same SPINE functionality both in TinyOS and in Ember ZigBee. In Table 3, the increase in code size is evaluated by using Equation (1). In the equation, (AG-EA) represents the size of automatically generated application code and (MW-EA) represents the size of manually written application code. Code size increments due to the automation process vary from 4% to 13% for both platforms.

(1) $$\begin{array}{ll} \text{Increment} & =\frac{((AG-EA)-(MW-EA))*100}{MW-EA}\\ AG & =\text{Automatically}\;\text{Generated}\\ MW & =\text{Manually}\;\text{Written}\\ EA & =\text{Empty}\;\text{Application} \end{array}$$

The advantage of using our framework, as opposed to manually writing the WSN application code, is that after the framework successfully generated correct target code for both platforms (*i.e.*, after we completed the initial debugging for our framework, using the simple token ring application), it took only two weeks for one person to implement the fully complex application (SPINE) described in Section 4. In other words, with our framework we could truly concentrate on spending the time modeling and simulating at the functional level, and then code generation, compilation and execution for two very different platforms was automated and extremely fast.

## 8. Conclusions

We have described an extensible framework for platform independent application modeling and hybrid simulation for sensor network algorithms based on MathWorks tools. The reason for choosing the MathWorks tools over, for example, TOSSIM, NS, OMNeT++, is that they are widely known and used, both in academia and in industry, and already provide rich libraries for digital signal processing and control algorithm behavior simulation. They also provide extensible mechanisms for efficient code generation and platform-specific retargeting. Possible extensions of this work include providing more library functions to support a broader variety of sensors and platforms, as well as improving the fidelity of the channel model, e.g., by considering more in detail the effects of congestion, noise, obstacles, distance and so on.

## Figures and Tables

**Table 1.** Simulation time for the token-ring network.

**Number of Nodes**	**Node Type**	**Simulation Time (s)**
8	Network	551
	SIM	0.36 (avg. each)
	HIL_SENS	256.87 (avg. each)
	HIL_RF	0.34 (avg. each)
	HIL_FULL	16.89 (avg. each)
16	Network	2069
	SIM	0.50 (avg. each)
	HIL_SENS	257.04 (avg. each)
	HIL_RF	0.46 (avg. each)
	HIL_FULL	257.00 (avg. each)
32	Network	4175
	SIM	0.91 (avg. each)
	HIL_SENS	258.28 (avg. each)
	HIL_RF	0.91 (avg. each)
	HIL_FULL	258.08 (avg. each)
64	Network	8396
	SIM	1.96 (avg. each)
	HIL_SENS	259.15 (avg. each)
	HIL_RF	1.97 (avg. each)
	HIL_FULL	259.13 (avg. each)

**Table 2.** Code size and memory usage for the token-ring application.

**Software System**	**Memory**	**Platform Base Code with Libs no Application Code (Bytes)**	**Automatic Code Generation (Bytes)**
TinyOS	ROM RAM	16,366	17,958
		840	918
Ember ZigBee	ROM RAM	87,326	89,662
		2738	2790

**Table 3.** Code size comparison between TinyOS and Ember ZigBee for SPINE.

**Software System**	**Memory**	**Platform Base Code with Libs no Application Code (Bytes)**	**Manual Impl. (Bytes)**	**Automatic(Bytes)**	**Increment**
TinyOS	ROM	9366	19,850	20,814	9.19%
	RAM	840	1355	1380	4.8%
Ember ZigBee	ROM	80,101	90,714	92,187	13.97%
	RAM	2736	3171	3220	11.26%
